# How Does Government Efficiency Affect Health Outcomes? The Empirical Evidence from 156 Countries

**DOI:** 10.3390/ijerph19159436

**Published:** 2022-08-01

**Authors:** Yemin Ding, Lee Chin, Fangyan Li, Peidong Deng

**Affiliations:** 1 School of Business and Economics, Universiti Putra Malaysia, Serdang 43400, Selangor, Malaysia; gs58281@student.upm.edu.my; 2 Jiangxi Institute of Regional Development, Jiangxi University of Technology, Nanchang 330098, China; fangyanli0706@163.com; 3 School of Economics and Finance, Xi’an Jiaotong University, Xi’an 710061, China; 18966708326@163.com

**Keywords:** government efficiency, health outcomes, heterogeneity analysis, mechanism analysis, moderating effect

## Abstract

This paper uses the unbalanced panel data of 156 countries during the period of 2002 to 2018 to explore the possible impact of government efficiency on health outcomes. Firstly, we used the fixed-effect model to examine the relationship between government efficiency and health outcomes and found that the increase in government efficiency can significantly improve health outcomes. Then, a series of robustness checks were carried out, which confirmed the reliability of the above result. Thirdly, this paper conducted a heterogeneity analysis from the perspective of life cycle. Fourthly, this paper investigated the mechanisms of the impact of government efficiency on health outcomes from the perspectives of economic growth, health innovation, education and corruption control. Finally, this paper studied the moderating effects of the ruling party’s ideology and democracy on the relationship between government efficiency and health outcomes. The findings of this study provide some references for governments to improve health outcomes.

## 1. Introduction

Agenda 21 for Sustainable Development of the United Nations points out that human health is the foundation of all social progress and development [1], which can be partially reflected in the systematic damage of COVID-19 to the economies of various countries [2]. After the outbreak of COVID-19, governments of various countries showed great differences in the efficiency of responding to the epidemic [3]. However, an objective fact is that, compared with the countries with low efficiency in the prevention and control of COVID-19, the countries that were able to quickly respond to the epidemic and carry out reasonable prevention and control measures tended to have a lower infection rate [4], which inspired us to consider whether government efficiency (according to Zheng [5], government efficiency refers to the quality and quantity of public services provided by governments per unit of time) is a factor that can affect health outcomes. Although researchers have conducted extensive and in-depth research on the influencing factors of health outcomes from the perspectives of economic factors, social factors and micro individual characteristics [6], there is still no literature on the possible impact of government efficiency on health outcomes. As such, this study fills the gap of empirical research in this field.

Government efficiency may affect health outcomes from the following aspects. Firstly, the improvement of government efficiency may directly improve health outcomes. In order to reasonably allocate health resources and effectively solve medical problems, governments of various countries have formulated a series of public health policies [7]. Wen et al. [8] believed that the effective implementation of policies is the basis for the policies to play their role, which is related to government efficiency. Supporting this, Glotko et al. [9] found that if a government is inefficient, which may be due to the inaction and power rent-seeking of the government staff during the implementation of policies, the implementation of policies will be blocked. As such, efficient governments are more likely to successfully implement public health policies, thereby promoting the improvement of health outcomes. Secondly, the improvement of government efficiency may affect health outcomes by affecting economic growth. According to Alam et al. [10], efficient governance can stimulate economic growth by promoting the effectiveness of labor division, the productivity of investment and the timeliness of social and economic policies. Supporting this, Alam et al. [10] used the system GMM to dynamically estimate the panel data of 81 countries and empirically confirmed the positive relationship between government efficiency and economic growth. Economic growth can promote the improvement of health outcomes in two ways. On the one hand, with the continuous improvement of a country’s economic level, the public health expenditure brought by the medical system reform tends to increase year by year, which can play a positive role in improving health outcomes [11]. On the other hand, economic growth is often accompanied by the increase in residents’ income, which can promote the growth of residents’ expenditure on health care and thus improve health outcomes [12]. As such, the improvement of government efficiency may improve health outcomes by promoting economic growth. Thirdly, the improvement of government efficiency may affect health outcomes by promoting health innovation. According to Wen et al. [8], the improvement of government efficiency can improve innovation by optimizing the institutional environment. Accordingly, as a kind of innovation, health innovation should also be improved with the improvement of government efficiency. Health innovation may affect health outcomes in two ways. On the one hand, health innovation, such as new therapeutic drugs with better efficacy and more advanced medical equipment, provides greater possibilities for curing patients, which has a positive impact on health outcomes [13]. On the other hand, the cost of health innovation is often transferred to patients, which is mainly reflected in the increase in medical price, which will increase the medical burden on patients and thus produce a negative impact on health outcomes [14]. As such, government efficiency may affect health outcomes by influencing health innovation, but the impact direction is uncertain. Fourthly, government efficiency may affect health outcomes by affecting education. According to Fomba et al. [15], the improvement of government efficiency can promote the improvement of education level by promoting the virtue and continuing education of teachers. Education can help to improve health outcomes by cultivating citizens’ healthy living habits [16]. As such, the improvement of government efficiency may improve health outcomes by promoting education. Finally, the improvement of government efficiency may affect health outcomes by curbing corruption. According to White [17], the resolute implementation of anti-corruption regulations is the key to effectively combating corruption, and whether anti-corruption regulations can be resolutely implemented to a large extent depends on government efficiency. In other words, the improvement of government efficiency can curb corruption by promoting the strong execution of anti-corruption regulations. However, the impact of anti-corruption on health outcomes is controversial. On the one hand, the mitigation of corruption can improve health outcomes by increasing the efficient use of medical funds [18] and the effectiveness of public health policies [19]. On the other hand, perhaps because anti-corruption reduces the motivation of government officials to provide the society with an appropriate number of high-quality public goods [20], Hessami [21] and Liang and Mirelman [18] found a positive relationship between corruption and government health expenditure, which means that less corruption may lead to less government health expenditure and thus produce a negative impact on health outcomes. As such, government efficiency may affect health outcomes by influencing anti-corruption, but the influence direction is not clear.

In order to empirically test the possible impact of government efficiency on health outcomes, we firstly used the fixed-effect model and the system GMM model to estimate the unbalanced panel data of 156 countries from 2002 to 2018, where the system GMM model is helpful to relieve potential endogenous problems. Secondly, motivated by the finding of Abegunde et al. [22] that the health risks and medical needs of residents of different ages are different, we further investigated the impact of government efficiency on the health of residents of different ages from the perspective of life cycle. Thirdly, as discussed above, government efficiency may not only directly affect health outcomes, but also indirectly affect health outcomes through economic growth, health innovation, education and curbing corruption. As such, this study explores the mechanism of the impact of government efficiency on health outcomes from the four perspectives of economic growth, health innovation, education and corruption control. Finally, motivated by the finding of Wen et al. [23] that different political parties pay different attention to public health, and the conclusion reached by Baqir [24] that governments with different degrees of democracy pay different attention to public health, we studied the moderating effects of the ideology of the party in power and the degree of democracy on the relationship between government efficiency and health outcomes.

The remaining part of the paper is organized as follows. Section 2 reviews the relevant literature. Section 3 describes the data and methodology. Empirical findings are reported and discussed in Section 4. Section 5 concludes and makes some policy implications.

## 2. Literature Review

The existing literature on the factors affecting health outcomes is mainly carried out from the perspectives of economy, environmental pollution, education and public health expenditure. From the perspective of economy, firstly, the relationship between economic growth and health outcomes is discussed. Although most of the literature recognizes the significant positive impact of economic growth on health outcomes [25,26], some studies reached different conclusions [12,27]. For example, by investigating the panel data of 27 European countries during the period of 2003 to 2014, Spiteri and von Brockdorff [12] found that the relationship between economic growth and health outcomes is not simply linear but inverted U-shaped. Secondly, the relationship between income inequality and health outcomes has also been found in some of the literature. Almost all the literature acknowledges that income inequality is an important factor that deteriorates the health of residents [28,29,30]. Barr [31] explained the negative impact of income inequality on health outcomes from the perspective of physiology. Barr [31] believed that income inequality, as a repeated stressor, can improve the allostatic load in human bodies for a long time, which will cause irreversible damage to blood vessels and other body tissues. Thirdly, urbanization can affect health outcomes by changing the urban environment and people’s living habits [32]. Compared with rural areas, cities have better medical resources [33]. As such, the status of health outcomes in the areas with a higher urbanization rate is often better [34]. Supporting this, Brueckner [35] confirmed the negative relationship between urbanization and mortality through a series of empirical studies. However, Chen et al. [36] pointed out that the impact of urbanization on health outcomes is not linear but inverted U-shaped. Finally, with the continuous deterioration of the contradiction between supply and demand in the global labor market, the possible negative impact of unemployment on health outcomes has aroused researchers’ concern [37,38] because the loss of good jobs may lead to “desperate death” of the unemployed [39]. Supporting this, McGee and Thompson [40] and Venkataramani et al. [41] empirically confirmed the positive impact of unemployment on disease and suicide. However, Wheaton [42] pointed out that the impact of unemployment on health may not always be negative, which depends on the pressure of the lost jobs.

The impact of environmental pollution on health outcomes seems to be uncontroversial. Almost all studies believe that pollution is a trigger of a large number of diseases and can significantly increase hospitalization and mortality [43,44,45,46]. As documented by Manisalidis et al. [47], about 9 million people die each year from environmental pollution.

Some of the literature explored the possible impact of education on health outcomes. On the one hand, education can develop people’s decision-making ability, promoting people to make decisions that are more conducive to health and improving the efficient use of health inputs [48]. On the other hand, education can develop people’s rational thinking, which is helpful in alleviating people’s irrational anxiety and consequently promoting health [49]. Supporting this, Adams [50], Van Kippersluis et al. [51] and Kemptner et al. [52] empirically confirmed the prominent role of education in improving health outcomes. However, several studies disputed this [53,54]. For example, Albouy and Lequien [53] explored a French longitudinal dataset by employing a non-parametric regression discontinuity design and a parametric two-stage approach and found that education has no significant impact on the improvement of health outcomes.

According to the health capital theory, public health investment plays a significant role in improving health outcomes [55]. Motivated by the theory, some of the literature empirically examines the relationship between public health expenditure and health outcomes and draw different conclusions [55]. Some studies found that public health expenditure can significantly improve health outcomes [55,56,57,58]. However, perhaps because of corruption, public health expenditure cannot always be used efficiently, which means that some public health resources are wasted [59]. As such, the increase in public health expenditure is not always effective in improving health outcomes. Supporting this, Gupta et al. [60] empirically found that the impact of public health expenditure on health outcomes is not significant. Moreover, Fayissa and Gutema [61] pointed out that the increase in public health expenditure significantly reduced life expectancy.

By combing the existing literature, we can find that up to now, there is no literature that explores health outcomes from the perspective of government efficiency. As such, we used the unbalanced panel data of 156 countries from 2002 to 2018 to explore the possible impact of government efficiency on health outcomes so as to fill the gap in this research field. If the impact of government efficiency on health outcomes is empirically confirmed, the finding can provide a new reference for governments on how to improve health outcomes.

## 3. Data and Methodology

The unbalanced panel data (limited to the availability of data, the attrition rate of the unbalanced panel data is 26.7%) used in this study involved 156 countries from 2002 to 2018. The list of the countries is presented in the List A1 of the Appendix A. Table 1 shows the descriptive statistics of all the variables involved in this study, including data sources. Additionally, in order to exclude the possibility of the estimation bias caused by multicollinearity, we conducted a multicollinearity test on the independent variables involved in this study, and the results are shown in Table 2. According to Sinan and Alkan [62], a VIF value less than 10 means that there is no multicollinearity. Referring to Table 2, we can find that the VIF values held by all the variables are less than 10, which means that the dataset used in this study did not have serious multicollinearity. The variables are further discussed below.

### 3.1. Dependent Variable

**Health outcomes (*Health*)**: The purpose of this study is to explore the impact of government efficiency on health outcomes. *Health* is the dependent variable in this study. Health outcomes represent how healthy a country is. Although health outcomes reflect the physical and mental well-being of residents within a community, this study focuses on physical health of residents. According to Gianino et al. [63], Disability-Adjusted Life Years (DALYs) is a credible comprehensive indicator that can be used to measure physical health outcomes. DALYs simultaneously measures the loss of life due to early death and that due to disability from disease [64]. In other words, DALYs measures both the incidence rate and the disease-related mortality. As such, compared with the single indicators, such as incidence rate and mortality, DALYs can more comprehensively reflect the health outcomes of a country. It should be emphasized that since DALYs measures the loss of life, a greater estimate of DALYs means worse health outcomes. Following Chang et al. [65], we selected DALYs as the proxy variable of *Health*.

### 3.2. Independent Variable

**Government efficiency (*Govefficiency*)**: *Govefficiency* is the main variable of interest in this study. According to Wen et al. [8], the estimate of government effectiveness jointly researched and released by the World Bank, Natural Resource Governance Institute and Brookings Institution can measure government efficiency from the three dimensions of political independence, public service quality and government credibility. As such, following Chang et al. [66] and Wen et al. [8], we used the estimate of government effectiveness as the proxy of *Govefficiency*, which can be obtained from the World Governance Indicators (WGI). The estimate of government effectiveness ranges from −2.5 to 2.5, and a higher estimate represents a more efficient government [66]. As discussed earlier, government efficiency may affect health outcomes in five ways: 1. direct impact, 2. economic growth, 3. innovation in health, 4. education and 5. corruption control. Importantly, the directions by which government efficiency affects health outcomes by affecting health innovation and corruption control are vague. As such, the direction of the impact of government efficiency on health outcomes is uncertain, which needs a series of empirical tests to confirm.

### 3.3. Control Variables

It should be emphasized again before explaining the control variables that the proxy variable of *Health* was DALYs, which measures the loss of life. As such, a higher DALYs means worse health outcomes. Therefore, if a variable has a positive impact on improving health outcomes, the variable should obtain a negative coefficient, not a positive one. Similarly, when a variable can worsen health outcomes, the variable should obtain a positive coefficient, not a negative one.

**Economic growth (*GDP*)**: According to Chen et al. [27], countries with more developed economies tend to have more developed medical systems and citizens with higher health awareness. As such, health outcomes in more developed areas should be better. Supporting this, Biggs et al. [25] empirically confirmed the positive impact of economic growth on health outcomes. As such, we added *GDP* into the model as a control variable and expected it to obtain a negative estimation coefficient. Following Aghion et al. [67], we used per capita gross domestic product to measure economic growth.

**Proportion of urban population in total population (*Urbanization*)**: Compared with rural areas, the medical resources in cities are relatively richer and better [33]. As such, regions with a higher urbanization level tend to have better health outcomes [34], which is consistent with the empirical results of Brueckner [35]. However, Hotez [68] pointed out that unhealthy eating habits and lack of exercise are also accompanied by the development of urbanization, which has a negative impact on health. As such, *Urbanization* was included in the analysis framework as a control variable, but the sign of its estimated coefficient was uncertain.

**CO_2_ emissions (*Air pollution*)**: Air pollution does great harm to both acute and chronic human health [69], which has been confirmed by extensive literature [70]. As such, we added *Air pollution* as a control variable into the model and expected it to obtain a positive estimation coefficient. There are many indicators to measure air pollution, such as PM2.5, PM10 and CO_2_ emissions. According to the availability of data, we chose CO_2_ emissions as the proxy variable of *Air pollution*.

**Proportion of unemployment in total labor force (*Unemployment*)**: According to Ardito et al. [71], unemployment can negatively affect the health of the unemployed by increasing their psychological burden. Supporting this, McGee and Thompson [40] and Venkataramani et al. [41] empirically confirmed the positive impact of unemployment on disease and suicide. As such, *Unemployment* was included in the analysis framework as a control variable and was expected to obtain a positive estimation coefficient.

**Proportion of current health expenditure in GDP (*Health expenditure*)**: The health capital theory holds that the increase in public health expenditure can significantly improve health outcomes [55], which has been empirically confirmed by Boachie and Ramu [57]. As such, *Health expenditure* was added into the model as a control variable and was expected to obtain a negative estimation coefficient. Following Wen et al., [23], we selected the proportion of current health expenditure in GDP as the proxy variable of *Health expenditure*.

**Gross secondary school enrollment rate (*Education*)**: Education can promote health by cultivating people’s healthy living habits [16] and reducing people’s irrational anxiety [49]. Supporting this, Kemptner et al. [52] empirically confirmed the prominent role of education in improving health outcomes. As such, we included *Education* as a control variable in the model and expected it to obtain a negative estimation coefficient. Following Wen et al. [8], we used gross secondary school enrollment rate as the proxy variable of *Education*.

**Estimate of control of corruption (*Corruption control*)**: On the one hand, corruption control can improve health outcomes by increasing the efficiency of public health expenditure [59]. On the other hand, anti-corruption reduces the motivation of government officials to provide an appropriate number of high-quality medical services to the society, which has a negative impact on health outcomes [20]. As such, *Corruption control* was included in the analysis framework as a control variable, but the sign of its estimation coefficient was not clear. Referring to Qu et al. [72], we used the estimate of control of corruption reported by World Governance Indicators (WGI) as the proxy variable of *Corruption control*. The estimate of control of corruption ranges from −2.5 to 2.5, and a higher estimate represents a cleaner government.

### 3.4. Methodology

The unbalanced panel data of 156 countries from 2002 to 2018 were collected to empirically investigate the possible impact of government efficiency on health outcomes. In order to reduce the influence of heteroscedasticity, the three variables *Health*, *GDP* and *Air pollution* were taken as logarithms. In addition, due to the data of *Govefficiency* and *Corruption control* ranging from negative to positive, this study upscaled the data of *Govefficiency* and *Corruption control* to positive values to ease the interpretation of the coefficients. Finally, we set up a panel data model as presented in Equation (1).

(1) $$Health_{it}=\alpha_{0}+\alpha_{1}{\mathrm{Govefficiency}}_{it}+\beta Z_{it}+\mu_{i}+\nu_{t}+\varepsilon_{it}$$

In Equation (1), *Z* represents a series of control variables that have been discussed in detail. $\mu_{i}$ and $\nu_{t}$ are the fixed effect for country and year, respectively. $\varepsilon_{it}$ is the error term, and $\alpha_{0}$, $\alpha_{1}$ and β are the coefficients to be estimated.

To ensure the robustness of the benchmark estimation results, a series of robustness checks were conducted. The placebo test is a robustness check that we planned to carry out first. Due to the undetected limitations in the research design, the effect of government efficiency on health outcomes reported by the benchmark estimation results may have been only a placebo effect. As such, once the effect of government efficiency on health outcomes was confirmed by the benchmark estimation results, the placebo test needed to be implemented to exclude the placebo effect. Refering to Cornaggia and Li [73], we first took out the data of *Govefficiency* of all samples, and then randomly assigned these data to each sample. Finally, we re-estimated Equation (1). If the effect of government efficiency on health outcomes indicated by benchmark estimation results was only a placebo effect, *Govefficiency* in the placebo test should obtain a statistically significant coefficient with the same sign as that obtained in the benchmark estimation.

Although the fixed-effect model can provide us with a range of reliable static estimation results, the static estimation ignores the endogeneity that may result from the correlation between government efficiency and health outcomes, which may lead to biased estimation results. As such, in order to alleviate the endogeneity, following Arellano and Bond [74], as presented in Equation (2), we added the lagged value of the dependent variable (*Health_i,t_*_−1_) as an instrumental variable into Equation (1) and then dynamically estimated Equation (2) using System GMM. In Equation (2), *Health_i,t−1_* is the lagged value of the dependent variable, and the other variables have the same meaning as in Equation (1).

(2) $$Health_{it}=\alpha_{0}+\alpha_{1}Health_{i,t-1}+\alpha_{2}Govefficiency_{it}+\beta Z_{it}+\varepsilon_{it}$$

Since the data of bureaucracy quality published by the International Country Risk Guide (ICRG) contain a variety of information, such as the rationality of the level setting of government officials, the effectiveness of the structure of government employees and the efficiency of administrative approval, bureaucracy quality was considered to be an alternative proxy variable for government efficiency [8]. As such, we changed the proxy variable of *Govefficiency* from the estimate of government effectiveness to bureaucracy quality and re-estimated Equation (1) for a robustness check. The data of bureaucracy quality are from the database of the International Country Risk Guide (ICRG).

## 4. Empirical Findings and Discussion

### 4.1. Benchmark Estimation Results

We first used the fixed-effect model to estimate Equation (1), and the estimation results are presented in Columns I and II of Table 3. Different from Column I, which ignores the control variables, Column II contains all the control variables. According to Columns I and II of Table 3, we can find that *Govefficiency* obtained a statistically significant negative coefficient regardless of whether the control variables were included, which indicates that the improvement of government efficiency can reduce DALYs. In other words, the increase in government efficiency can significantly improve health outcomes. As for the control variables, the signs held by all the control variables were as expected with at least 10% significant level. Firstly, the estimated coefficients of *GDP*, *Urbanization* and *Unemployment* showed that economic development plays an important positive role in improving health outcomes. Specifically, the statistically significant negative coefficient held by *GDP* and *Urbanization* indicated that economic growth and urbanization can significantly reduce DALYs, that is, can significantly improve health outcomes, which is consistent with the findings of Biggs et al. [25] and Brueckner [35]. The statistically significant positive coefficient held by *Unemployment* indicated that an increase in the unemployment rate can increase DALYs. In other words, a difficult employment situation is a reason for deteriorating health outcomes, which has been confirmed by McGee and Thompson [40] and Venkataramani et al. [41]. As such, in order to promote the improvement of health outcomes, governments should commit themselves to economic development aimed at high growth, high urbanization rate and low unemployment rate. Secondly, *Air pollution* obtained a positive coefficient, which was significant at the 1% significance level, indicating that clean air is the foundation of health. Thirdly, the statistically significant negative coefficient obtained by *Education* indicated that the popularization of secondary education can significantly decrease DALYs, that is, can significantly improve health outcomes. Finally, *Corruption control* obtained a negative coefficient, which was significant at the 1% significance level, indicating that a clean government is an important guarantee for people’s health. Supporting this, Wen et al. [75] found that corruption is a huge cancer that hinders social development and welfare reform.

### 4.2. Robustness Checks

In order to ensure the robustness of the benchmark estimation results, a series of robustness checks were carried out, and the results are shown in Columns III to VIII of Table 3, in which the singular columns ignore the control variables, while the dual columns contain the control variables. Columns III and IV report the estimated results of the placebo test. The results show that the coefficients obtained by *Govefficiency* in Columns III and IV were not statistically significant, which excludes the possibility that the positive effect of government efficiency on improving health outcomes derived from the benchmark estimations was only a placebo effect. To alleviate the potential endogeneity, following Arellano and Bond [74], we used System GMM to dynamically estimate the relationship between government efficiency and health outcomes, and the estimation results are shown in Columns V and VI. According to the results, it can be found that the Sargan value was not significant at the 10% significance level, which indicates that there is no over-identification problem from the instrumental variable. Additionally, AR(1) was significant at the 1% significance level, while AR(2) was not significant at the 10% significance level, which shows that there is no auto-correlation problem from error term. According to Columns V and VI, we can find that, consistent with the benchmark estimation results, *Govefficiency* obtained a statistically significant negative coefficient, which indicated that the negative effect of government efficiency on DALYs also existed in the dynamic estimation. In other words, the positive effect of government efficiency on improving health outcomes also existed in the dynamic estimation. According to Columns VII and VIII, we can find that, after the proxy variable of *Govefficiency* was changed from the estimate of government effectiveness to bureaucracy quality, *Bureaucracy quality* obtained a statistically significant negative coefficient, which once again proved the robustness of the negative relationship between government efficiency and DALYs, that is, the increase in government efficiency can significantly improve health outcomes.

### 4.3. Further Analysis

#### 4.3.1. Heterogeneity Analysis

Motivated by the finding of Abegunde et al. [22] that the health risks and medical needs of residents of different ages are different, from the perspective of life cycle, this study further investigated the effect of government efficiency on the health of residents of different ages. In addition to the overall data of DALYs for all ages (DALYs All ages), the World Health Organization (WHO) also published the data of DALYs for different age groups, namely DALYs 0–4, DALYs 5–14, DALYs 15–49, DALYs 50–69 and DALYs 70+. As such, we changed the proxy variable of *Health* from DALYs All ages to DALYs 0–4, DALYs 5–4, DALYs 15–49, DALYs 50–69 and DALYs 70+, in turn, and then re-estimated Equation (1) for heterogeneity analysis. The estimated results of the heterogeneity analysis are reported in Table 4. According to Table 4, we can find that, consistent with the benchmark estimation results, *Govefficiency* in Columns I, IV and V obtained a statistically significant negative coefficient, which indicated that the improvement of government efficiency can significantly reduce the DALYs of residents aged 0–4 and over 50. In other words, the improvement of government efficiency can play a significant positive role in improving the health of residents aged 0–4 and over 50. However, the statistically insignificant coefficients held by *Govefficiency* in Columns II and III indicate that the improvement of government efficiency has no significant effect on reducing DALYs of residents aged 5 to 49. In other words, the improvement of government efficiency cannot significantly improve the health of residents aged 5 to 49. This discrepancy in the impact of government efficiency on the health of residents of different ages may be explained from two aspects. On the one hand, compared with children and young adults, infants and the elderly are more susceptible to diseases due to their sensitivity to viruses and relatively weak immunity [22]. On the other hand, the income-generating ability of infants and the elderly is weak, which leads to their medical problems requiring additional attention from governments. As such, government efficiency has a more significant impact on the health of residents aged 0 to 4 and over 50.

As for the control variables, first of all, compared with the residents of the other four age groups (DALYs 0–4, DALYs 5–14, DALYs 15–49 and DALYs 50–69), *GDP* and *Health expenditure* both had the strongest effect on reducing DALYs 70+. In other words, economic growth and public health expenditure have the strongest positive effect on improving the health of people over 70 years old. This may also be because the elderly are more vulnerable to diseases, and their income level is relatively low [22]. Specifically, the elderly have a relatively greater risk of infection, and their own ability to bear medical expenses is relatively weak. As such, compared with other groups, the elderly are more in need of public health expenditure to cope with their potential disease risk. Therefore, public health expenditure has the strongest effect on reducing DALYs of residents over 70 years old. As for *GDP*, since high economic level is an important guarantee for sufficient public health expenditure, *GDP* also had the strongest effect on reducing DALYs of residents over 70 years old. Secondly, surprisingly, the coefficients obtained by *Air pollution* in Columns I and V were statistically insignificant, which indicated that air pollution has no significant impact on the health of infants and the elderly. This may be because infants and the elderly are neither school-aged nor in the working population. As such, when air pollution is serious, infants and the elderly can choose not to go out to avoid the negative impact of air pollution on their health. In addition, the statistically significant positive coefficient obtained by *Unemployment* in Column V indicated that the increase in the unemployment rate can significantly increase the DALYs of people over 70 years old, that is, the increase in the unemployment rate has a significant negative impact on the health of residents over 70 years old. However, interestingly, for the residents under 14 years old, who also belong to the non-labor population like the people over 70 years old, the increase in the unemployment rate had no significant impact on their DALYs (DALYs 0–4 and DALYs 5–14). The reason for this difference may stem from the human sense of responsibility for the next generation. Specifically, according to Sainsbury et al. [76], when children are ill, parents are willing to do their best to restore their children to health, although the parents’ economic situation is not very optimistic. On the contrary, few children are willing to spend all they have to pay good medical care for their parents when their elderly parents are infected with serious diseases. As such, when a difficult employment environment leads to the decline of the income level of the working population, they may spend less on their elderly parents’ medical care, which can lead to the deterioration of the health status of the elderly. This reminds the young adults to take more responsibility for supporting their parents in the family [12].

#### 4.3.2. Mechanism Analysis

In this section, we further explore how government efficiency affects health outcomes. As discussed earlier, in addition to directly affecting health outcomes, government efficiency may also affect health outcomes through economic growth, health innovation, education and corruption control. Referring to Wen et al. [8], we empirically tested the mechanism of the impact of government efficiency on health outcomes from the perspectives of economic growth, health innovation, education and corruption control by adding interactions into Equation (1). Specifically, as shown in Equation (3), we added the interaction of *Govefficiency* and *GDP* into Equation (1) to test whether government efficiency can affect health outcomes by affecting economic growth. As shown in Equation (4), we added the interaction of *Govefficiency* and *Health innovation* into Equation (1) to test whether government efficiency can affect health outcomes by affecting health innovation. There are two perspectives to measure health innovation, namely, health innovation input and output. Due to the availability of data, we chose the proportion of domestic R&D expenditure on health in GDP as the proxy variable of *Health innovation* from the perspective of health innovation input. The data of *Health innovation* can be obtained from World Health Organization (WHO). It must be emphasized that, due to the strong correlation between *Health expenditure* and *Health innovation*, we had to delete *Health expenditure* from the control variables when estimating Equation (4) to avoid biased estimation results caused by multicollinearity. As shown in Equation (5), we added the interaction of *Govefficiency* and *Education* into Equation (1) to test whether government efficiency can affect health outcomes by affecting education. As shown in Equation (6), we added the interaction of *Govefficiency* and *Corruption control* into Equation (1) to test whether government efficiency can affect health outcomes by affecting anti-corruption. In Equations (3)–(6), all variables have the same meaning as in Equation (1).

(3) $$Health_{it}=\alpha_{0}+\alpha_{1}Govefficiency_{it}+\alpha_{2}Govefficiency_{it}\times GDP_{it}+\beta Z_{it}+\mu_{i}+\nu_{t}+\varepsilon_{it}$$

(4) $$Health_{it}=\alpha_{0}+\alpha_{1}Govefficiency_{it}+\alpha_{2}Govefficiency_{it}\times Health\;innovation_{it}+\beta Z_{it}+\mu_{i}+\nu_{t}+\varepsilon_{it}$$

(5) $$Health_{it}=\alpha_{0}+\alpha_{1}Govefficiency_{it}+\alpha_{2}Govefficiency_{it}\times Education_{it}+\beta Z_{it}+\mu_{i}+\nu_{t}+\varepsilon_{it}$$

(6) $$Health_{it}=\alpha_{0}+\alpha_{1}Govefficiency_{it}+\alpha_{2}Govefficiency_{it}\times Corruptioncontrol_{it}+\beta Z_{it}+\mu_{i}+\nu_{t}+\varepsilon_{it}$$

We used the fixed-effect model to estimate Equations (3)–(6), and the estimated results are shown in Table 5. According to Table 5, we can find that *GDP* in Column I, *Health innovation* in Column II, *Education* in Column III and *Corruption control* in Column IV all obtained a statistically significant negative coefficient, which indicated that economic growth, health innovation, education and corruption control all contribute to the reduction in DALYs, that is, economic growth, health innovation, education and corruption control can significantly improve health outcomes. At the same time, the interaction terms of *Govefficiency* × *GDP* in Column I, *Govefficiency* × *Health innovation* in Column II, *Govefficiency* × *Education* in Column III and *Govefficiency* × *Corruption control* in Column IV all also obtained a statistically significant negative coefficient, which indicated that the improvement of government efficiency can decrease DALYs by promoting economic growth, health innovation, education and anti-corruption. In other words, the increase in government efficiency can improve health outcomes by promoting economic growth, health innovation, education and anti-corruption.

#### 4.3.3. Moderating Effects

The government’s attention to different fields may be influenced by the ideology of the ruling party because the party in power has the motivation to give more attention and support to the industries where they can obtain support votes when seeking re-election. As such, the ideology of the party in power may affect the government’s attention to the medical industry [23] and thus have a moderating effect on the relationship between government efficiency and health outcomes. Specifically, according to Qiu et al. [77], left-wing parties prefer to support labor-intensive industries, while right-wing parties tend to pay more attention to capital-intensive industries. As such, as a capital-intensive industry, the medical industry is more likely to be focused on by a right-wing government [78]. Based on the above analysis, we believed that the ideology of the ruling party may have a moderating effect on the impact of government efficiency on health outcomes, and when a right-wing party is in power, government efficiency should have a stronger effect on improving health outcomes. In order to empirically test the moderating effect of the ruling party’s ideology, we divided the full sample into two subsamples, namely, left-wing governments and right-wing governments, and then used Equation (1) to estimate the two subsamples, respectively. Following Cotoc et al. [79], we obtained the information on the ideology of governments from the IDB’s Database of Political Institutions (DPI). The estimated results of the moderating effect of the ruling party’s ideology are shown in Columns I and II of Table 6. According to the results, we can find that the coefficient obtained by *Govefficiency* in Column I was not statistically significant, which shows that when the left-wing party is in power, the improvement of government efficiency cannot significantly reduce DALYs, that is, the increase in government efficiency cannot significantly improve health outcomes. However, *Govefficiency* in Column II obtained a statistically significant negative coefficient, which indicated that when the right-wing party is in power, the improvement of government efficiency can significantly reduce DALYs, that is, the increase in government efficiency can significantly improve health outcomes. These empirical results support the above analysis, that is, the ideology of the party in power has a moderating effect on the relationship between government efficiency and health outcomes, and when the right-wing party is in power, the improvement of government efficiency can more significantly improve health outcomes.

According to Baqir [24], democracy promotes the political participation of the poor, which increases the possibility for governments to consider the needs of the poor. As the poor are more likely to face health problems and thus fall into a poverty trap, quality health care has become an important demand for the poor [80]. As such, in order to get more support votes from the poor, governments tend to give more attention and support to public health, such as allocating a higher share of the public budget to public health [24]. Based on the above analysis, we believed that the degree of democracy may have a moderating effect on the impact of government efficiency on health outcomes, and in more democratic countries, government efficiency should play a more positive role in improving health outcomes. In order to test the moderating effect of democracy, we divided the full sample into two subsamples, namely, low democratic countries and high democratic countries, and then used Equation (1) to estimate the two sub-samples, respectively. According to Jetter et al. [81], the variable of *Polity2* in the Polity IV project provided us with data on the degree of democracy of countries around the world. The data of *Polity2* range from −10 to 10, and a higher value represents a more democratic country. As such, we classified countries with a value of *Polity2* less than 0 as low democratic countries and classified countries with a value of *Polity2* greater than 0 as high democratic countries. The estimated results of the moderating effect of democracy are presented in Columns III and IV of Table 6. According to the results, we can find that, although the improvement of government efficiency can reduce the DALYs of both low democratic countries and high democratic countries, this effect was stronger in high democratic countries. These empirical results support the above analysis, that is, democracy has a moderating effect on the relationship between government efficiency and health outcomes, and the improvement of government efficiency can improve health outcomes more significantly in more democratic countries.

## 5. Conclusions

This paper used a fixed-effect model, placebo test and dynamic GMM estimation to investigate the impact of government efficiency on health outcomes in 156 countries from 2002 to 2018. At the same time, per capita GDP, urbanization, CO_2_ emissions and other indicators that may affect health outcomes were included in the analysis framework as control variables. The empirical results showed that the improvement of government efficiency can significantly reduce DALYs, that is, the increase in government efficiency can significantly improve health outcomes. Then, this study performed a heterogeneity analysis from the perspective of life cycle. Specifically, we examined the effect of government efficiency on the health of residents of five different age groups and found that the increase in government efficiency can significantly improve the health of the residents aged 0 to 4 and over 50 but cannot significantly affect the health of the residents aged 5 to 49. Thirdly, this study explored the mechanism of the effect of government efficiency on health outcomes from the perspectives of economic growth, health innovation, education and corruption control and found that the increase in government efficiency can improve health outcomes by promoting per capita GDP, the investment in health innovation, the popularization of secondary education and anti-corruption. Finally, this paper examined the moderating effects of the ruling party’s ideology and democracy on the relationship between government efficiency and health outcomes, and the two moderating effects were confirmed by the empirical results. Specifically, when the right-wing party is in power and a country is more democratic, the increase in government efficiency can more significantly improve health outcomes.

The findings of this study provide some references for governments to promote the improvement of health outcomes. Firstly, in view of the inhibitory effect of government efficiency on DALYs, governments should further improve their efficiency. According to Wen et al. [8], highly educated government staff, a good administrative system and service-oriented government are the characteristics of an efficient government. As such, governments can improve their efficiency by improving the educational level of employees, optimizing the institutional environment and building a service-oriented government. Secondly, based on the finding that the increase in government efficiency has a more significant effect on improving the health of people under the age of 4 and over the age of 50, which may be because infants and the elderly have a greater risk of infection with diseases and because the income-generating ability of infants and the elderly is weak, governments should pay more attention to the health problems of infants and the elderly. Thirdly, based on the finding that government efficiency has no significant impact on health outcomes when the left-wing party is in power, we suggest that left-wing parties should pay more attention to the medical industry when they are in power in addition to the labor-intensive industries where they can obtain support votes. Finally, in view of the positive moderating effect of democracy on the positive impact of government efficiency on health outcomes, we suggest that governments should continue to promote the process of democratization, promoting people’s political participation.

## Figures and Tables

**Table 1:** Descriptive statistics of full sample.

Variable Name	Measurement	Mean	Standard Deviation	Min	Max	Source
Health	Estimate	39,650.99	19,393.06	15,517.19	1.87 × 10^5^	World Health Organization (WHO)
Govefficiency	Estimate	0.01	1.00	−2.48	2.44	World Governance Indicators (WGI)
GDP	Constant 2015 USD	15,021.01	22,137.57	258.63	1.71 × 10^5^	World Development Indicator (WDI)
Urbanization	Ratio	0.58	0.24	0.09	1.00	World Development Indicator (WDI)
Air pollution	Metric tons per capita	4.48	5.46	0.00	47.70	World Development Indicator (WDI)
Unemployment	Ratio	0.08	0.06	0.00	0.37	World Development Indicator (WDI)
Health expenditure	Ratio	0.06	0.03	0.01	0.24	World Development Indicator (WDI)
Education	Ratio	0.82	0.29	0.06	1.64	World Development Indicator (WDI)
Corruption control	Estimate	0.01	1.00	−1.87	2.47	World Governance Indicators (WGI)

**Table 2:** Multicollinearity test.

Variable Name	VIF	1/VIF
Govefficiency	9.37	0.106752
Corruption control	8.18	0.1222
GDP	3.38	0.296236
Education	3.33	0.299889
Urbanization	2.6	0.384623
Air pollution	2.26	0.441658
Health expenditure	1.82	0.548074
Unemployment	1.27	0.788695
Mean VIF	4.03	

**Table 3:** Baseline regression results and robustness checks.

	Fixed Effect		Placebo Test		SYS-GMM		Variable Replacement	
	I	II	III	IV	V	VI	VII	VIII
Lagged dep. var					0.967 ***	1.020 ***		
					(170.69)	(104.93)		
Govefficiency	−0.036 ***	−0.024 ***	0.001	0.001	−0.017 ***	−0.006 ***		
	(−3.28)	(−3.03)	(0.64)	(0.87)	(−8.77)	(−3.08)		
Bureaucracy quality							−0.068 ***	−0.059 ***
							(−5.10)	(−3.34)
GDP		−0.028 *		−0.015		−0.011 *		−0.051 ***
		(−1.74)		(−0.92)		(−1.95)		(−3.09)
Urbanization		−0.617 ***		−0.678 ***		0.102 ***		−0.089
		(−7.11)		(−7.77)		(6.43)		(−0.92)
Air pollution		0.067 ***		−0.070 ***		0.014 ***		−0.014
		(5.96)		(−6.20)		(3.46)		(−0.97)
Unemployment		0.317 ***		−0.308 ***		−0.044 *		−0.372 ***
		(3.18)		(−3.06)		(−1.77)		(−3.17)
Health expenditure		−0.685 ***		−0.593 ***		−0.178 ***		−1.368 ***
		(−3.04)		(−2.60)		(−2.58)		(−5.28)
Education		−0.161 ***		−0.155 ***		−0.003		−0.137 ***
		(−5.34)		(−5.10)		(−0.48)		(−4.43)
Corruption control		−0.057 ***		−0.039 ***		0.003		−0.013
		(−5.52)		(−4.34)		(1.18)		(−1.26)
Constant	10.464 ***	11.259 ***	10.465 ***	11.206 ***	0.339 ***	−0.179	10.614 ***	11.292 ***
	(8525.35)	(94.54)	(8587.66)	(95.25)	(5.75)	(−1.38)	(346.48)	(92.77)
Sargan test					0.631	0.317		
AR (1)					0.000	0.001		
AR (2)					0.621	0.265		
R-squared	0.003	0.214	−0.065	0.143			0.016	0.141
country FE	YES	YES	YES	YES			YES	YES
year FE	YES	YES	YES	YES			YES	YES

Notes: t-statistics in parenthesis; ***, ** and * indicate statistical significance at the 1%, 5% and 10% levels, respectively.

**Table 4:** Heterogeneity analysis.

	DALYs 0–4	DALYs 5–14	DALYs 15–49	DALYs 50–69	DALYs 70+	DALYs All Ages
	I	II	III	IV	V	VI
Govefficiency	−0.041 **	−0.008	−0.009	−0.024 ***	−0.022 ***	−0.024 ***
	(−2.45)	(−0.82)	(−0.86)	(−2.81)	(−3.50)	(−3.03)
GDP	−0.134 ***	−0.105 ***	−0.122 ***	−0.116 ***	−0.675 ***	−0.028 *
	(−8.79)	(−6.85)	(−8.31)	(−9.78)	(−23.28)	(−1.74)
Urbanization	−1.208 ***	−0.973 ***	−0.814 ***	−0.782 ***	−0.433 ***	−0.617 ***
	(−7.79)	(−11.90)	(−9.93)	(−9.92)	(−6.85)	(−7.11)
Air pollution	−0.003	0.024 **	0.040 ***	0.021 **	−0.016	0.067 ***
	(−0.17)	(2.33)	(4.82)	(2.00)	(−1.50)	(5.96)
Unemployment	−0.102	−0.097	0.223 **	0.340 ***	0.588 ***	0.317 ***
	(−1.09)	(−1.08)	(2.37)	(4.69)	(3.30)	(3.18)
Health expenditure	−0.835 ***	−0.639 ***	−1.116 ***	−0.852 ***	−2.639 ***	−0.685 ***
	(−3.93)	(−3.00)	(−5.45)	(−5.19)	(−6.54)	(−3.04)
Education	−0.495 ***	−0.238 ***	−0.085 ***	−0.031	−0.001	−0.161 ***
	(−9.17)	(−8.36)	(−2.99)	(−1.15)	(−0.03)	(−5.34)
Corruption control	−0.051 ***	−0.040 ***	−0.053 ***	−0.030 ***	−0.012	−0.057 ***
	(−2.75)	(−4.09)	(−5.42)	(−3.18)	(−1.57)	(−5.52)
Constant	17.711 ***	10.961 ***	11.433 ***	12.509 ***	12.967 ***	11.259 ***
	(83.15)	(97.60)	(101.63)	(115.61)	(149.46)	(94.54)
R-squared	0.617	0.433	0.252	0.265	0.199	0.214
country FE	YES	YES	YES	YES	YES	YES
year FE	YES	YES	YES	YES	YES	YES

Notes: t-statistics in parenthesis; ***, ** and * indicate statistical significance at the 1%, 5% and 10% levels, respectively.

**Table 5:** Mechanism analysis.

	Economic Growth	Health Innovation	Education	Corruption Control
	I	II	III	IV
Govefficiency	−0.154 ***	−0.044 ***	−0.058 **	−0.033 ***
	(−2.63)	(−2.70)	(−2.18)	(−2.94)
GDP	−0.025 **	−0.087 ***	−0.034 **	−0.025
	(−2.41)	(−4.48)	(−2.08)	(−1.52)
Urbanization	−0.615 ***	−0.453 ***	−0.634 ***	−0.617 ***
	(−7.09)	(−3.12)	(−7.33)	(−7.12)
Air pollution	−0.068 ***	0.005	0.066 ***	0.065 ***
	(−5.96)	(0.27)	(5.90)	(5.66)
Unemployment	−0.313 ***	0.456 ***	0.330 ***	0.329 ***
	(−3.12)	(4.17)	(3.32)	(3.29)
Health expenditure	−0.688 ***		−0.661 ***	−0.703 ***
	(−3.05)		(−2.94)	(−3.12)
Health innovation		−0.144 ***		
		(−2.61)		
Education	−0.161 ***	−0.060	−0.144 ***	−0.166 ***
	(−5.33)	(−1.54)	(−4.73)	(−5.48)
Corruption control	−0.057 ***	0.006	−0.053 ***	−0.057 ***
	(−5.49)	(0.34)	(−5.18)	(−5.55)
Govefficiency × GDP	−0.015 **			
	(−2.15)			
Govefficiency × Health Innovation		−0.131 **		
		(−1.98)		
Govefficiency × Education			−0.117 ***	
			(−3.87)	
Govefficiency × Corruption control				−0.018 *
				(−1.70)
Constant	11.259 ***	11.597 ***	11.322 ***	11.278 ***
	(94.50)	(71.56)	(94.61)	(94.37)
R-squared	0.214	0.209	0.221	0.217
country FE	YES	YES	YES	YES
year FE	YES	YES	YES	YES

Notes: t-statistics in parenthesis; ***, ** and * indicate statistical significance at the 1%, 5% and 10% levels, respectively.

**Table 6:** Moderating effect analysis.

	Ideology of the Party in Power		Degree of Democracy	
	Left-Wing Governments	Right-Wing Governments	Low Democratic Countries	High Democratic Countries
	I	II	III	IV
Govefficiency	−0.002	−0.028 **	−0.002 ***	−0.064 ***
	(−0.20)	(−2.47)	(−3.88)	(−3.19)
GDP	−0.068 ***	−0.006	−0.024	−0.057 ***
	(−5.25)	(−0.23)	(−0.80)	(−4.48)
Urbanization	−0.252 ***	−0.840 ***	−0.813 ***	−0.343 ***
	(−3.64)	(−5.67)	(−5.45)	(−4.45)
Air pollution	0.017 *	0.142 ***	−0.100 ***	0.047 ***
	(1.88)	(6.88)	(−5.10)	(4.84)
Unemployment	0.424 ***	0.181	−0.397	−0.264 ***
	(6.58)	(0.73)	(−1.43)	(−4.06)
Health expenditure	0.149	−0.638 *	−0.858 **	−0.546 **
	(0.65)	(−1.96)	(−2.52)	(−2.39)
Education	−0.074 ***	−0.234 ***	−0.219 ***	0.046
	(−2.89)	(−4.77)	(−4.26)	(1.61)
Corruption control	−0.037 ***	−0.075 ***	−0.039 **	−0.030 ***
	(−4.31)	(−4.33)	(−2.24)	(−3.47)
Constant	11.229 ***	11.198 ***	11.335 ***	11.076 ***
	(109.47)	(58.64)	(54.48)	(102.33)
R-squared	0.203	0.312	0.294	0.156
country FE	YES	YES	YES	YES
year FE	YES	YES	YES	YES

Notes: t-statistics in parenthesis; ***, ** and * indicate statistical significance at the 1%, 5% and 10% levels, respectively.

## Data Availability

The data used in this study are available on request from the corresponding author.

## Appendix A

### List A1: The Country List

Albania, Algeria, Andorra, Angola, Antigua and Barbuda, Argentina, Armenia, Australia, Austria, Azerbaijan, Bahrain, Bangladesh, Barbados, Belarus, Belgium, Belize, Benin, Bermuda, Bhutan, Bolivia, Bosnia and Herzegovina, Botswana, Brazil, Bulgaria, Burkina Faso, Burundi, Cambodia, Cameroon, Canada, Cape Verde, Central African Republic, Chad, Chile, China, Colombia, Comoros, Cook Islands, Costa Rica, Croatia, Cuba, Cyprus, Denmark, Djibouti, Dominica, Dominican Republic, Ecuador, El Salvador, Equatorial Guinea, Eritrea, Estonia, Ethiopia, Finland, France, Gabon, Georgia, Germany, Ghana, Greece, Grenada, Guatemala, Guinea, Guinea-Bissau, Guyana, Haiti, Honduras, Hungary, Iceland, India, Indonesia, Iraq, Ireland, Israel, Italy, Japan, Jordan, Kazakhstan, Kenya, Kiribati, Kuwait, Latvia, Lebanon, Lesotho, Liberia, Lithuania, Luxembourg, Madagascar, Malawi, Malaysia, Maldives, Mali, Malta, Mauritania, Mauritius, Mexico, Moldova, Mongolia, Morocco, Mozambique, Myanmar, Namibia, Nepal, Netherlands, New Zealand, Nicaragua, Niger, Nigeria, North Macedonia, Norway, Oman, Pakistan, Panama, Paraguay, Peru, Philippines, Poland, Portugal, Puerto Rico, Qatar, Romania, Rwanda, Samoa, Saudi Arabia, Senegal, Serbia, Seychelles, Sierra Leone, Singapore, Slovenia, Solomon Islands, Somalia, South Africa, Spain, Sri Lanka, Sudan, Sweden, Tajikistan, Tanzania, Thailand, Togo, Tonga, Trinidad and Tobago, Tunisia, Turkey, Turkmenistan, Tuvalu, Uganda, Ukraine, United Arab Emirates, United Kingdom, United States, Uruguay, Uzbekistan, Vanuatu, Vietnam, Zambia, Zimbabwe.

## References

[1] Jiang T., Deng Z., Zhi Y., Cheng H., Gao Q. (2021). The effect of urbanization on population health: Evidence from China. Front. Public Health.

[2] Coccia M. (2021). The relation between length of lockdown, numbers of infected people and deaths of COVID-19, and economic growth of countries: Lessons learned to cope with future pandemics similar to COVID-19 and to constrain the deterioration of economic system. Sci. Total Environ..

[3] Martínez-Córdoba P.J., Benito B., García-Sánchez I.M. (2021). Efficiency in the governance of the COVID-19 pandemic: Political and territorial factors. Glob. Health.

[4] Alanezi F., Aljahdali A., Alyousef S.M., Alrashed H., Mushcab H., AlThani B., Alghamedy F., Alotaibi H., Saadah A., Alanzi T. (2020). A comparative study on the strategies adopted by the United Kingdom, India, China, Italy, and Saudi Arabia to contain the spread of the COVID-19 pandemic. J. Healthc. Leadersh..

[5] Pratap P., Dickson A., Love M., Zanoni J., Donato C., Flynn M.A., Schulte P.A. (2021). Public health impacts of underemployment and unemployment in the United States: Exploring perceptions, gaps and opportunities. Int. J. Environ. Res. Public Health.

[6] Greer S.L., Bekker M.P.M., Azzopardi-Muscat N., McKee M. (2018). Political analysis in public health: Middle-range concepts to make sense of the politics of health. Eur. J. Public Health.

[7] Wen J., Deng P., Zhang Q., Chang C. (2021). Is higher government efficiency bringing about higher innovation?. Technol. Econ. Dev. Econ..

[8] Glotko A.V., Polyakova A.G., Kuznetsova M.Y. (2020). Main trends of government regulation of sectoral digitalization. Entrep. Sustain. Issues.

[9] Zheng J. (1992). Dictionary of International Relations.

[10] Alam M.R., Kitenge E., Bedane B. (2017). Government effectiveness and economic growth. Econ. Bull..

[11] Vu T.V. (2020). Economic complexity and health outcomes: A global perspective. Soc. Sci. Med..

[12] Spiteri J., von Brockdorff P. (2019). Economic development and health outcomes: Evidence from cardiovascular disease mortality in Europe. Soc. Sci. Med..

[13] Datta S., Barua R., Das J. (2020). Application of artificial intelligence in modern healthcare system. Alginates—Recent Uses of This Natural Polymer.

[14] Murray C.J., Abbafati C., Abbas K.M., Abbasi M., Abbasi-Kangevari M., Abd-Allah F., Nagaraja S.B. (2020). Five insights from the global burden of disease study 2019. Lancet.

[15] Fomba B.K., Talla D.N.D.F., Ningaye P. (2022). Institutional Quality and Education Quality in Developing Countries: Effects and Transmission Channels. J. Knowl. Econ..

[16] Hahn R.A., Truman B.I. (2015). Education improves public health and promotes health equity. Int. J. Health Serv..

[17] White L.D. (1955). Introduction to the Study of Public Administration.

[18] Liang L., Mirelman A.J. (2014). Why do some countries spend more for health? An assessment of sociopolitical determinants and international aid for government health expenditures. Soc. Sci. Med..

[19] Montes G.C., Paschoal P.C. (2015). Corruption: What are the effects on government effectiveness? Empirical evidence considering developed and developing countries. Appl. Econ. Lett..

[20] Lichand G., Lopes M.F.M., Medeiros M.C. (2016). Is Corruption Good For Your Health? Harvard University, Pontifical Catholic University. https://scholar.harvard.edu/glichand/publications/job-market-paper.

[21] Hessami Z. (2014). Political corruption, public procurement, and budget composition: Theory and evidence from OECD countries. Eur. J. Polit. Econ..

[22] Abegunde D.O., Mathers C.D., Adam T., Ortegon M., Strong K. (2007). The burden and costs of chronic diseases in low-income and middle-income countries. Lancet.

[23] Wen J., Deng P., Fu Q., Chang C. (2021). Does health innovation relieve disease burden? The comprehensive evidence. Technol. Forecast. Soc. Change.

[24] Baqir R. (2002). Social Sector Spending in a Panel of Countries. IMF Working Paper No. 02/35. https://www.imf.org/external/pubs/ft/wp/2002/wp0235.pdf.

[25] Biggs B., King L., Basu S., Stuckler D. (2010). Is wealthier always healthier? The impact of national income level, inequality, and poverty on public health in Latin America. Soc. Sci. Med..

[26] Qiu Y., Chen X., Shi W. (2020). Impacts of social and economic factors on the transmission of coronavirus disease 2019 (COVID-19) in China. J. Popul. Econ..

[27] Chen X., Shao S., Tian Z., Xie Z., Yin P. (2017). Impacts of air pollution and its spatial spillover effect on public health based on China’s big data sample. J. Clean. Prod..

[28] Kawachi I., Kennedy B.P. (1997). The relationship of income inequality to mortality: Does the choice of indicator matter?. Soc. Sci. Med..

[29] Massing M.W., Rosamond W.D., Wing S.B., Suchindran C.M., Kaplan B.H., Tyroler H.A. (2004). Income, income inequality, and cardiovascular disease mortality: Relations among county populations of the United States, 1985 to 1994. South. Med. J..

[30] Singer B., Ryff C.D. (2001). The influence of inequality on health outcomes. New Horizons in Health: An Integrative Approach.

[31] Barr D.A. (2014). Health Disparities in the United States: Social Class, Race, Ethnicity, and Health.

[32] Li X., Song J., Lin T., Dixon J., Zhang G., Ye H. (2016). Urbanization and health in China, thinking at the national, local and individual levels. Environ. Health.

[33] Moore M., Gould P., Keary B.S. (2003). Global urbanization and impact on health. Int. J. Hyg. Environ. Health.

[34] Liu G., Sun M., Wang Z., Jian W. (2016). Association analysis between urbanization and non-communicable diseases and health-related behavior. J. Peking Univ. Health Sci..

[35] Brueckner M. (2019). Adult mortality and urbanization: Examination of a weak connection in sub-Saharan Africa. World Dev..

[36] Chen H., Liu Y., Li Z., Xue D. (2017). Urbanization, economic development and health: Evidence from China’s labor-force dynamic survey. Int. J. Equity Health.

[37] Benach J., Vives A., Amable M., Vanroelen C., Tarafa G., Muntaner C. (2014). Precarious employment: Understanding an emerging social determinant of health. Annu. Rev. Public Health.

[38] Roelfs D.J., Shor E., Davidson K.W., Schwartz J.E. (2011). Losing life and livelihood: A systematic review and meta-analysis of unemployment and all-cause mortality. Soc. Sci. Med..

[39] Case A., Deaton A. (2015). Rising morbidity and mortality in midlife among white non-Hispanic Americans in the 21st century. Proc. Natl. Acad. Sci. USA.

[40] McGee R.E., Thompson N.J. (2015). Unemployment and depression among emerging adults in 12 states, behavioral risk factor surveillance system, 2010. Prev. Chronic Dis..

[41] Venkataramani A.S., Bair E.F., O’Brien R.L., Tsai A.C. (2020). Association between automotive assembly plant closures and opioid overdose mortality in the United States. JAMA Intern. Med..

[42] Wheaton B. (1990). Life Transitions, Role Histories, and Mental Health. Am. Sociol. Rev..

[43] Eze I.C., Schaffner E., Fischer E., Schikowski T., Adam M., Imboden M., Tsai M., Carballo D., von Eckardstein A., Künzli N. (2014). Long-term air pollution exposure and diabetes in a population-based Swiss cohort. Environ. Int..

[44] Hashim D., Boffetta P. (2014). Occupational and environmental exposures and cancers in developing countries. Ann. Glob. Health.

[45] Kan H., Chen R., Tong S. (2012). Ambient air pollution, climate change, and population health in China. Environ. Int..

[46] Kelishadi R., Poursafa P. (2010). Air pollution and non-respiratory health hazards for children. Arch. Med. Sci..

[47] Manisalidis I., Stavropoulou E., Stavropoulos A., Bezirtzoglou E. (2020). Environmental and health impacts of air pollution: A review. Front. Public Health.

[48] Lochner L. (2011). Non-Production Benefits of Education: Crime, Health, and Good Citizenship. NBER Working Paper No. 16722. https://www.nber.org/system/files/working_papers/w16722/w16722.pdf.

[49] Brunello G., Fort M., Schneeweis N., Winter-Ebmer R. (2015). The causal effect of education on health: What is the role of health behaviors?. Health Econ..

[50] Adams S.J. (2002). Educational attainment and health: Evidence from a sample of older adults. Educ. Econ..

[51] Van Kippersluis H., O’Donnell O., van Doorslaer E. (2011). Long run returns to education: Does schooling lead to an extended old age?. J. Hum. Resour..

[52] Kemptner D., Jürges H., Reinhold S. (2011). Changes in compulsory schooling and the causal effect of education on health: Evidence from Germany. J. Health Econ..

[53] Albouy V., Lequien L. (2009). Does compulsory education lower mortality?. J. Health Econ..

[54] Braakmann N. (2011). The causal relationship between education, health and health related behaviour: Evidence from a natural experiment in England. Int. J. Health Econ..

[55] Arthur E., Oaikhenan H.E. (2017). The effects of health expenditure on health outcomes in Sub-Saharan Africa (SSA). Afr. Dev. Rev..

[56] Anyanwu J.C., Erhijakpor A.E.O. (2009). Health expenditures and health outcomes in Africa. Afr. Dev. Rev..

[57] Boachie M.K., Ramu K. (2016). Effect of public health expenditure on health status in Ghana. Int. J. Health.

[58] Kamiya Y. (2010). Determinants of Health in Developing Countries: Cross-Country Evidence. OSIPP Discussion Paper No. DP-2010-E-009, Osaka School of International Public Policy (OSIPP). https://www.osipp.osaka-u.ac.jp/archives/DP/2010/DP2010E009.pdf.

[59] Azfar O., Gurgur T. (2008). Does corruption affect health outcomes in the Philippines?. Econ. Gov..

[60] Gupta S., Verhoeven M., Tiongson E.R. (2002). The effectiveness of government spending on education and health care in developing and transition economies. Eur. J. Polit. Econ..

[61] Fayissa B., Gutema P. (2005). Estimating a health production function for Sub-Saharan Africa (SSA). Appl. Econ..

[62] Sinan A., Alkan B.B. (2015). A useful approach to identify the multicollinearity in the presence of outliers. J. Appl. Stat..

[63] Gianino M.M., Savatteri A., Politano G., Nurchis M.C., Pascucci D., Damiani G. (2021). Burden of COVID-19: Disability-Adjusted Life Years (DALYs) across 16 European countries. Eur. Rev. Med. Pharmacol. Sci..

[64] Prüss-Üstün A., Campbell-Lendrum D., Corvalán C., Woodward A. (2003). The Global Burden of Disease Concept. WHO Environmental Burden of Disease Series.

[65] Chang A.Y., Skirbekk V.F., Tyrovolas S., Kassebaum N.J., Dieleman J.L. (2019). Measuring population ageing: An analysis of the global burden of disease study 2017. Lancet Public Health.

[66] Chang C.P., Wen J., Zheng M., Dong M., Hao Y. (2018). Is higher government efficiency conducive to improving energy use efficiency? Evidence from OECD countries. Econ. Model..

[67] Aghion P., Alesina A.F., Trebbi F. (2007). Democracy, Technology, and Growth.

[68] Hotez P.J. (2017). Global urbanization and the neglected tropical diseases. PLoS Negl. Trop. Dis..

[69] Kan H., Wu T.C. (2013). Ambient air pollution, and human health in China: The past and future. Acad. J. Second Mil. Med. Univ..

[70] Cai J., Zhao A., Zhao J., Chen R., Wang W., Ha S., Xu X., Kan H. (2014). Acute effects of air pollution on asthma hospitalization in Shanghai, China. Environ. Pollut..

[71] Ardito C., Leombruni R., Mosca M., Giraudo M., d’Errico A. (2017). Scar on my heart: Effects of unemployment experiences on coronary heart disease. Int. J. Manpow..

[72] Qu G., Slagter B., Sylwester K., Doiron K. (2019). Explaining the standard errors of corruption perception indices. J. Comp. Econ..

[73] Cornaggia J., Li J.Y. (2019). The value of access to finance: Evidence from M&As. J. Financ. Econ..

[74] Arellano M., Bond S.R. (1991). Some tests of specification for panel data: Monte Carlo evidence and an application to employment equations. Rev. Econ. Stud..

[75] Wen J., Zheng M., Feng G.F., Chen S.W., Chang C.P. (2020). Corruption and innovation: Linear and nonlinear investigations of OECD countries. Singap. Econ. Rev..

[76] Sainsbury C.P., Gray O.P., Cleary J., Davies M.M., Rowlandson P.H. (1986). Care by parents of their children in hospital. Arch. Dis. Child..

[77] Qiu Q., Nian Y.J., Guo Y., Tang L., Lu N., Wen L.Z., Liu K.J. (2019). Development and validation of three machine-learning models for predicting multiple organ failure in moderately severe and severe acute pancreatitis. BMC Gastroenterol..

[78] Ye C., Ni X. (2020). Medical insurance governance modernization and medical supply side reform: A review and revelation of the foreign frontier research literature. Soc. Sci. Abroad.

[79] Cotoc I., Johri A., Sosa-Padilla C. (2021). Sovereign Spreads and the Political Leaning of Nations. NBER Working Papers No. 29197. https://www.nber.org/system/files/working_papers/w29197/w29197.pdf.

[80] Shadmi E., Chen Y., Dourado I., Faran-Perach I., Furler J., Hangoma P., Willems S. (2020). Health equity and COVID-19: Global perspectives. Int. J. Equity Health.

[81] Jetter M., Agudelo A.M., Hassan A.R. (2015). The effect of democracy on corruption: Income is key. World Dev..

